# Concurrent Improvement in Maize Grain Yield and Nitrogen Use Efficiency by Enhancing Inherent Soil Productivity

**DOI:** 10.3389/fpls.2022.790188

**Published:** 2022-04-14

**Authors:** Zhigang Wang, Bao-Luo Ma, Yajian Li, Rongfa Li, Qi Jia, Xiaofang Yu, Jiying Sun, Shuping Hu, Julin Gao

**Affiliations:** ^1^College of Agronomy, Inner Mongolia Agricultural University, Hohhot, China; ^2^Ottawa Research and Development Centre, Agriculture and Agri-Food Canada, Ottawa, ON, Canada; ^3^Tongliao Academy of Agricultural Sciences, Tongliao, China

**Keywords:** maize, inherent soil productivity, yield, nitrogen use efficiency, sustainable agriculture

## Abstract

Quantifying the relationships of maize yield and nitrogen use efficiency (NUE) to inherent soil productivity (ISP) could provide a theoretical basis for implementing strategies that concurrently narrow the yield gap, increase NUE, and improve soil quality. A field study under irrigation conditions was conducted at five locations with large differences in ISP (estimated by maize grain yield without using fertilizers) across various ecological regions in Inner Mongolia, China. Our results showed that the changes in maize yield and nitrogen partial factor productivity (PFP_N_) to ISP followed a linear-plateau model, with a common inflection point. When ISP was below 8.0 t ha^–1^, maize yield and PFP_N_ were stagnating at a low level, due to a trade-off between the contribution of soil and chemical fertilizers to yield. When ISP exceeded 8.0 t ha^–1^, the contribution rate of soil to yield stabilized at 80%, resulting in a simultaneous increase in maize yield by 1.2 t ha^–1^ and PFP_N_ by 4.6 kg kg^–1^, for increasing ISP at each t ha^–1^. Our results indicated that while keeping other management strategies unchanged, to increase maize yield and PFP_N_ by 15% or further 30%, it is necessary to increase ISP to 9.3 and 10.4 t ha^–1^, respectively. In this scenario, N input will be reduced by 33.5 and 47.5%, and apparent N losses will be reduced by 11.7 and 21.5%, respectively. Therefore, enhancing ISP could lead to a simultaneous improvement in yield and NUE as well as strongly support the green development of maize production.

## Introduction

Over the past five decades, the total grain production in China has increased by 5.4-fold. Yet, it is accompanied by a substantial increase in fertilizer consumption, accounting for 35% of the world fertilizer consumption, but with only 7% of the world’s farmland area (Fan et al., 2012). Excessive fertilization has reduced nitrogen use efficiency (NUE) of grain production in China to less than 30%, and caused a number of problems, such as increased production cost, agricultural non-point source pollution, and greenhouse gas emissions (Chen et al., 2011). Maize is the most productive crop in China, with an average NUE of only 30% (Chen et al., 2014). As the third largest maize producing province in China, the NUE of maize in Inner Mongolia is about 25%, which is approximately half that of the United States (Zhang et al., 2015). Efficient use of N inputs for concurrent improvements in grain yield and NUE has been recognized as a feasible strategy to promote sustainable agricultural development and environmental safety (Wang et al., 2014).

In China, the medium- and low-yielding farmland accounts for 70% of the total arable land (Fan et al., 2012). Additionally, long-term poor management has led to serious soil degradation problems. About 48.7 million hectare of farmland has degraded to varying degrees, accounting for 40% of the country’s arable land (Wang et al., 2019). In the past five decades, the average thickness of the topsoil has gradually decreased from 22.9 to 17.6 cm (Zhang et al., 2016), with an average soil organic matter (SOM) of only 10 g kg^–1^, which is far below the 25–40 g kg^–1^ SOM content in the European Union and the United States (Fan et al., 2012). These problems are the root causes of the declining soil productivity, excessive fertilizer input, and inefficient use of resources in crop production. Chen et al. (2011) demonstrated that integrated soil–crop system management (ISCSM) can help meet the growth of global food demand in 2030, while significantly reducing N fertilizer application, N loss, and greenhouse gas emissions. Here, soil improvement is a prerequisite for achieving the ISCSM goal. Under China’s Zero Fertilizer Use Growth Action Policy, exploring inherent soil productivity (ISP) potential is an inevitable choice to simultaneously increase crop yields and environmental safety (Lal, 2006; Johnston et al., 2009).

Inherent soil productivity is the internal capacity of soil to receive and store water and nutrients and create a favorable environment for crop production (Andrews and Carroll, 2001). Considering that it is a comprehensive index, such as the physical, chemical, biological, hydrological, and thermal properties of the soil, the ISP is usually evaluated by crop yield without using fertilizers (Fan et al., 2013). Previous studies have systematically addressed the relationship between soil productivity and crop yield through meta-analysis. For example, Pan et al. (2009) conducted an analytical study based on the SOM and crop yield data for the past 50 years. They found that soil productivity was positively correlated with field crop yield, and negatively correlated with yield variability, indicating that soil carbon sequestration could significantly improve crop yield and yield stability. However, they noticed that the weakening of these correlations in recent years indicated that the ISP was gradually decreasing. In contrast, Fan et al. (2013) assessed the historical change in ISP obtained from 7,410 on-farm trials and 8 long-term experiments conducted since 1980. They reported an increase in ISP (farm yield without fertilizer) by 0.73–1.76 t ha^–1^ since 1980s. Although the result of this historical increase also involved the contribution of cultivar improvement and plant density increase, it certainly shows the following key points. First, the higher the SOM content, the higher the ISP; second, the higher ISP, the higher yield of the best cultivated population and the lower the yield variability; third, the higher the yield of cultivated population, the greater the contribution of ISP to crop yield. On the one hand, these results illustrated the importance of the ISP improvement, especially increases in SOM content, to increase and stabilize crop yields. On the other hand, these results indicated that with the increase in ISPs, the demand for water and fertilizer required to achieve a certain yield target may be considerably reduced, resulting in a significant improvement in resource use efficiency.

Few studies have, however, examined the relationship between ISP and crop NUE, and existing conclusions are not always consistent. Zeng et al. (2012) observed that compared with those in low ISP fields, paddy rice in high ISP fields accumulated more N and exhibited higher N partial factor productivity (PFP_N_) but with lower NUE. Therefore, they concluded that there was a luxury N uptake by paddy rice in high ISP fields. Dong et al. (2020) found no significant difference in NUE among various ISP levels in paddy rice production. Jin et al. (2014) found that by improving the SOM and total N content to increase the ISP in low ISP farmlands, the annual yield and water use efficiency (WUE) of winter wheat-summer maize system increased by 8 and 9.5%, respectively, whereas there was no significant change in NUE. Zhang et al. (2011) showed that N input for maximum yield and maximum NUE in paddy rice production was relatively low in high ISP farmlands compared with those in medium- and low-ISP farmlands. They suggested that increasing ISP could greatly reduce N input. An et al. (2015) observed that high ISP fields have significantly reduced N loss per unit N input and remarkably increased rice yields, compared with low ISP fields. In addition, rice yield in low ISP fields could not reach the level of high ISP fields, even with high N input, because of the significant increase in the observed loss of reactive N (Yang G. Y. et al., 2020).

Enhancing ISP could positively affect crop yield and N efficiency. However, limited available research has focused on large-scale analysis and on small grain cereal crops, such as rice and wheat. There is a lack of quantitative assessment of the relationship between ISP and maize yield and/or between ISP and NUE. Therefore, the present study conducted a large-scale network field experiment at five locations with various ISPs. Using maize yield and NUE data obtained in different site-year environments, we quantitatively analyzed the relationship between ISP and maize yield and between ISP and NUE, and predicted the short-term and/or long-term goal for ISP enhancement. We hypothesized that enhancing ISP could simultaneously improve maize grain yield and NUE, while significantly reduce apparent N losses in maize production. Our objective was to provide a theoretical basis for concurrently narrowing the yield gap and increasing the N efficiency of maize by improving the ISP.

## Materials and Methods

### Experimental Site

To get various ISP conditions, a field experiment was carried out for three growing seasons (2014–2016) at five locations over a large area from the east to the west of Inner Mongolia, the People’s Republic of China. These locations represented the typical maize production regions in Inner Mongolia, i.e., Hilly Region of Southern Greater Khingan, West Liao River Plain, Hilly Region of Northern Yan Mountains, Tumed Plain, and Hetao Plain. At each location, the previous crop was always maize, and the field was all under conventional management, specifically with shallow rotary tillage, and without crop residue return and manure application. Soil texture, groundwater table, SOM, and other soil physical and chemical properties, such as available nutrients differed largely among locations, as shown in Table 1. Due to the differences in seasonal temperatures and possible effect on maize growth, a common hybrid Pioneer 335 was used at each location. Taking into account the significant differences in precipitation among locations, micro-sprinkler irrigation was only supplied when maize leaves were temporary withered, to eliminate the limitation of water deficit on crop growth (Table 1).

**TABLE 1:** Locations, soil fertility, and weather data from April to October of the multisite-year network experiment conducted in Inner Mongolia, China.

	Location							Soil fertility			
Ecological region	Latitude	Longitude	Soil texture	Groundwater table (m)	Year	Average temperature (°C)	Precipitation (mm)	Soil organic matter (g kg^–1^)	Available N (mg kg^–1^)	Olsen P (mg kg^–1^)	Available K (mg kg^–1^)
Hilly region of southern greater khingan	46°45′N	122°47′E	Sandy clay loam	6.6	2015	17.6	436.9	16.2	80.3	54.7	108.4
					2016	20.2	378.8	17.9	101.4	31.2	164.9
West liao river plain	43°44′N	122°32′E	Silty loam	14.1	2015	20.5	433.2	20.3	65.6	28.2	267.9
					2016	21.3	331.6	21.1	77.3	32.8	227.4
Hilly region of northern yan mountains	42°18′N	118°10′E	Sandy loam	12.8	2015	19.1	273.1	21.7	59.2	29.4	113.8
					2016	20.2	501.7	22.9	53.9	34.8	151.7
Tumed plain	40°32′N	110°28′E	Silty loam	6.3	2015	17.8	275.4	26.7	77.1	24.1	106.3
					2016	19.6	308.2	29.7	100.1	18.3	151.0
Hetao plain	41°11′N	108°49′E	Clay loam	4.7	2015	17.0	230.5	18.2	79.5	24.5	214.5
					2016	18.2	138.5	16.4	108.0	34.6	195.6

### Experimental Design

At each location, a three-factor factorial experiment (2 × 3 × 2) involving two soil management strategies [conventional practices (CPs) vs. improved soil productivity (IMSP)], three plant densities (60,000, 82,500, and 105,000 plants ha^–1^) and two fertilizer rates (0 and 220 kg N ha^–1^) was conducted. Adjacent to the CP strategy, additional control treatments without the use of chemical fertilizers were added to estimate the ISP of different plant densities at each location. The CP strategy was a conventional management method adopted by smallholder farmers, specifically with shallow rotary tillage to a depth of 15 cm, without crop residue return and manure application. The IMSP strategy was a practice aimed at effectively improving ISP. Its goal was to increase SOM by approximately 10 g kg^–1^ by applying 79.5 t ha^–1^ composted sheep manure (average organic matter content of 30%) along with returning maize crop residues and deep tillage to a depth of 35 cm. In the spring of the following year, the soil was prepared by compacting with a 10-cm shallow rotary tillage.

At each location, the factorial experiment was arranged in a split–split plot design with three replications. The soil management strategy was assigned in the main plots, plant density in the subplots, and fertilizer rate in the sub-subplots. Each sub-subplot contained 12 rows (60-cm interrow spacing) of maize and was 12-m in length. At planting, all treatments except the unfertilized control plot received 46 kg P ha^–1^ as triple superphosphate and 37 kg K ha^–1^ as potassium sulfate, as the starter fertilizer. It was banded at 5 cm below and 5 cm to the side of the seed row. For the 220 kg N ha^–1^ treatment, the applied N as urea was side-dressed at the 6-leaf stage, at a soil depth of about 10 cm, and 10 cm from the plant row to avoid the potential damage to maize seedlings while reducing reactive nitrogen losses (Zhang et al., 2009; Chen et al., 2011, 2014). An effective weed control was achieved with the use of pre-plant and post-emergence herbicides. Pesticides or fungicides were sprayed by unmanned aerial vehicle when needed.

In this study, the ISP of each location was estimated by using the average maize grain yields of different plant densities in the unfertilized control plots under CP management (Table 2). These control plots received neither organic amendments (i.e., manure and crop residues) nor chemical fertilizers during the 3-year experimental period. However, the same agronomic practices, such as herbicide application for weed control, insect and disease control measures, and supplemental irrigation were performed on all the plots.

**TABLE 2:** Inherent soil productivity and maize grain yield of treatments averaged across 2 years (2015 and 2016) at five locations in Inner Mongolia.

Locations		Hilly region of southern greater khingan	West liao river plain	Hilly region of northern yan	Hetao plain	Tumed plain
					
ISP		7.6	11.5	10.3	8.0	10.2
Soil management (S)	CP	9.3	14.8	10.4	11.2	11.4
	IMSP	10.3	15.1	12.1	12.3	13.1
Plant density (PD)	6.0	9.6	13.2	10.7	10.2	11.3
	8.25	10.2	14.8	11.3	13.0	12.1
	10.5	9.7	16.8	11.7	12.0	13.3
N Rate (N)	0	8.3	12.5	11.1	8.5	11.1
	220	9.8	14.9	11.3	11.8	12.3
Source of variance						
S		<0.0001	0.0001	0.0001	0.0482	0.0001
PD		NS	0.0001	<0.0001	0.0236	0.0002
N		0.0034	0.0001	0.0001	0.0021	0.0001
S × PD		NS	NS	NS	NS	<0.0001
S × N		NS	NS	0.0280	NS	NS
PD × N		NS	0.0001	NS	0.0424	0.0431
S × PD × N		NS	NS	0.0001	NS	0.0054

### Soil and Plant Sampling

Before land preparation, composite soil samples (0–30 cm depth) of each plot were taken with a soil auger in 3 points, and analyzed for SOM (organic C content determined by wet digestion with H_2_SO_4_–K_2_Cr_2_O_7_ and converted to SOM by multiplying by 1.724), alkali-hydrolyzable N, Olsen-P, NH_4_OAc-K, and pH. Before planting and after harvest, the soil NO_3_^–^-N and NH_4_^+^-N concentrations in the 0–90 cm depth were monitored with soil core sampler (3 cm inside diameter). Three cores per plot were taken and separated into 30 cm depth increments to form three composite samples (one for each depth) in each plot. Field moist soil samples were thoroughly mixed, and representative sub-samples were extracted immediately by shaking with 0.01 *M* CaCl_2_ solution (1:10 soil: solution ratio) for 1 h on a rotary shaker (180 rev min^–1^) followed by filtration. The extracts were directly analyzed for NO_3_^–^-N and NH_4_^+^-N concentrations by an automated continuous flow analyzer (SEAL AA3, Germany) or were stored at −19°C until they were analyzed by the same method within 3 months. The same soil samples were also used for the determination of soil bulk density and water content. The soil mineral N (N_min_, such as NO_3_^–^-N and NH_4_^+^-N) (kg N ha^–1^) was calculated by multiplying the NO_3_^–^-N and NH_4_^+^-N concentrations (mg kg^–1^ dry soil) by the soil bulk density of the different soil layers.

At the V6 stage, 20 consecutive and uniform plants in each plot were tagged for later biomass sampling. At physiological maturity (R6), five consecutive plants from the tagged area were cut at the stem base, separated into leaves, stalks (including stems, leaf sheaths, tassels, ear shanks, husks, and cobs) and grain. The chopped plant samples were dried at 85 ± 5°C to constant weight for recording the dry weights. Dried plant material and grain samples were ground to fine powder for the determination of N concentration, using the Kjeldahl method. The N content of each fraction was calculated as the product of N concentration by its biomass.

At physiological maturity (R6), after investigating the actual plant density, all ears from the central two rows of each plot were hand-harvested. Then, the ear was shelled and recorded for grain moisture and grain weight. Grain yield was calculated and reported on a 140 g kg^–1^ water basis.

### Data Analysis

To eliminate the residual nutrient effect and reasonably evaluate ISP, only the data of 2015 and 2016 were analyzed. After testing the homogeneity of the experimental errors at each site-year, we combined all datasets from the five locations and 2 years and used CurveExpert 1.4 (Hyams Development) to select the best fitting model with the highest *R*^2^. According to the analysis results, a linear-plateau regression model was used to fit the relationships between ISP and yield, and between ISP and N partial productivity, and a parabolic model was selected to fit the relationship between ISP and NUE. These analyses were performed in accordance with the NLIN procedure in SPSS 17.0 (SPSS Institute Inc., Chicago, IL, United States), and the corresponding critical inflection point of the model was determined by following the modified piecewise regression method (Passos et al., 2012). To examine the key determinants for ISP, the relationship between ISP and soil physico-chemical parameters was analyzed using the linear regression procedure of SPSS 17.0 software, and the coefficient of determination of linear regression (*R*^2^) was compared to quantitatively reflect the contribution of soil physico-chemical parameters to the change of ISP.

To eliminate the deviation induced by location and study year, the relative yield and relative contribution of soil and fertilization to grain yield were calculated as follows:


(1) $$\mathrm{Relative}\mathrm{yield}\left(\%\right)={\text{GY}}_{\text{T}}/{\text{GY}}_{\text{Max}}\times 100$$

where GY_T_ is the grain yield (kg ha^–1^ at 14% grain moisture) of any treatment in a given location, and GY_Max_ is the maximum yield of the fertilized treatment in the same location.


(2) $$\begin{array}{c} \mathrm{Relative}\mathrm{contribution}\mathrm{of}\mathrm{soil}\mathrm{to}\mathrm{yield}\left(\%\right)\\ ={\text{GY}}_{\mathrm{unfert}.}/{\text{GY}}_{\mathrm{fert}.}\times 100 \end{array}$$

(3) $$\begin{array}{c} \mathrm{Relative}\mathrm{contribution}\mathrm{of}\mathrm{fertilization}\mathrm{to}\mathrm{yield}\left(\%\right)\\ =\left({\text{GY}}_{\mathrm{fert}.}-{\text{GY}}_{\mathrm{unfert}.}\right)/{\text{GY}}_{\mathrm{fert}.}\times 100 \end{array}$$

where GY_unfert._ is the grain yield of unfertilized treatment, i.e., ISP, and GY_fert._ is the grain yield of a treatment receiving 220 kg N ha^–1^.

Nitrogen utilization parameters, such as PFP_N_ and NUE were calculated according to Wang et al. (2014) as follows:


(4) $${\text{PFP}}_{\text{N}}={\text{GY}}_{\mathrm{fert}.}/N\mathrm{fertilizer}\mathrm{applied}$$

(5) $$\text{NUE}=\left({\text{GY}}_{\mathrm{fert}.}-{\text{GY}}_{0N}\right)/N\mathrm{fertilizer}\mathrm{applied}$$

where GY_0*N*_ is the grain yield of 0 N treatment.

An N budget was estimated for evaluating the nutrient mineralization and retention capacity of the soil (Meng et al., 2012). Apparent soil N mineralization rate (N_mineralizable_) and apparent N loss (N_loss_) during the maize growth season were estimated by the balance of inputs and outputs of a given treatment according to the following formula:


(6) $$\begin{array}{c} N_{\text{mineralizable}}\left(\mathrm{kg}{\mathrm{ha}}^{-1}\right)\\ =N_{\mathrm{uptake}\left(\mathrm{ISP}\right)}+\mathrm{Residual}N_{\min\left(\mathrm{ISP}\right)}-\mathrm{Initial}N_{\min\left(\mathrm{ISP}\right)} \end{array}$$

where N_uptake(ISP)_ is the crop N uptake from the ISP treatment, Residual N_min(ISP)_ is the residual 0–90 cm soil N_min_ in the ISP treatment measured on samples taken after harvest, and Initial N_min(ISP)_ is the initial 0–90 cm soil N_min_ in the ISP treatment tested before planting.


(7) $$\begin{array}{c} {\text{N}}_{loss}\left(\mathrm{kg}{\mathrm{ha}}^{-1}\right)={\text{N}}_{\text{input}}+\mathrm{Initial}N_{\text{min}}\\ +{\text{N}}_{\text{mineralizable}}-{\text{N}}_{\text{uptake}}-\mathrm{Residual}N_{\text{min}} \end{array}$$

Where N_input_ is the N application rate, N_uptake_ is the crop N uptake from fertilized treatment under a given soil management strategy, Initial N_min_ is the initial 0–90 cm soil N_min_ in fertilized treatment tested before planting, and Residual N_min_ is the residual 0–90 cm soil N_min_ in fertilized treatment tested after harvesting.

Water use efficiency (WUE) of maize crop was determined by dividing the grain yield by water consumption during the growing season as follows:


(8) $$\mathrm{WUE}\left(\mathrm{kg}{\mathrm{ha}}^{-1}{\text{mm}}^{-1}\right)=\mathrm{GY}/\left(W_{p}-{\text{W}}_{\text{h}}+\text{P}+\text{I}\right)$$

where GY is maize grain yield (kg ha^–1^), W_*p*_ is the 0–90 cm soil water content (mm) at planting, W_*h*_ is the 0–90 cm soil water content (mm) at harvest, P is the growing season precipitation (mm), and I is the amount of irrigation (mm).

## Results

### Relationship Between Inherent Soil Productivity and Maize Grain Yield

**FIGURE 1:**
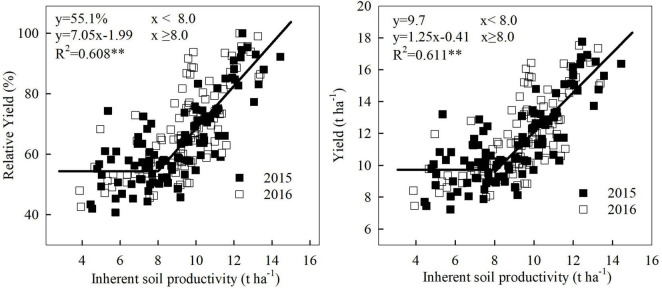
The relationship between maize grain yield and inherent soil productivity: Linear-plateau models based on the data from a multisite-year network experiment conducted at five locations across large regions from the east to the west of Inner Mongolia, China. **means significant at the level of *p* < 0.01.

Soil management strategy, plant density, and N rate, all significantly affected the maize grain yield at each location, but their interaction effects seemed to be site-specific (Table 2). The ISP differed significantly at the five experimental locations. In general, the higher ISP, the higher maize grain yield of treatment was observed. The dynamic change of maize yield with ISP exhibited two stages: fluctuation phase and linear increase phase, showing a common inflection point of ISP at 8.0 t ha^–1^ (Figure 1). When ISP was below 8.0 t ha^–1^, relative yield remained at approximately 55%, and the corresponding actual yield stagnated at around 9.7 t ha^–1^. Maize grain yield increased linearly and significantly when ISP exceeded 8.0 t ha^–1^. Specifically, relative yield and actual yield increased, respectively, by 7.1% and 1.2 t ha^–1^ for every 1 t ha^–1^ increase in ISP.

**FIGURE 2:**
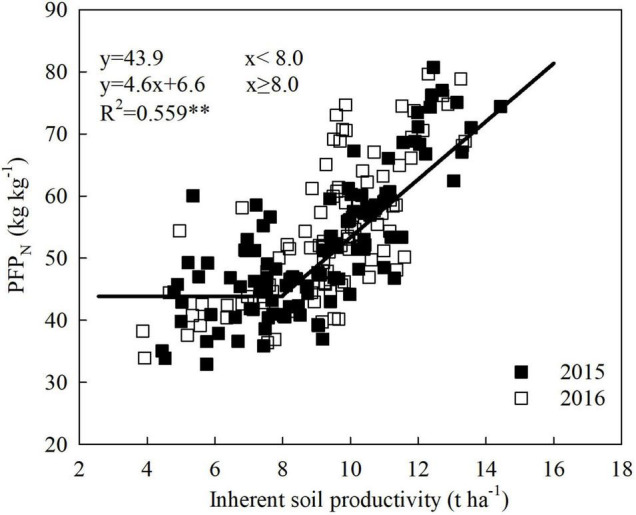
The relationship between N partial factor productivity (PFP_N_) of maize and inherent soil productivity: A linear-plateau model based on the data from a 2-year field experiment conducted at five locations across large regions from the east to the west of Inner Mongolia, China. **means significant at the level of *p* < 0.01.

Similar to the trend of maize yield, PFP_N_ of maize exhibited a plateau with linear increase along with ISP improvement, and the inflection point also appeared at ISP of 8.0 t ha^–1^ (Figure 2). With the same N input, PFP_N_ stayed at around 43.9 kg kg^–1^ when ISP was below 8.0 t ha^–1^. When ISP was above 8.0 t ha^–1^, PFP_N_ increased linearly. Specifically, PFP_N_ increased by 4.6 kg kg^–1^ for every 1 t ha^–1^ increase in ISP.

### Contribution of Soil and N Fertilizer to Maize Yield and Its Relationship With Inherent Soil Productivity

**FIGURE 3:**
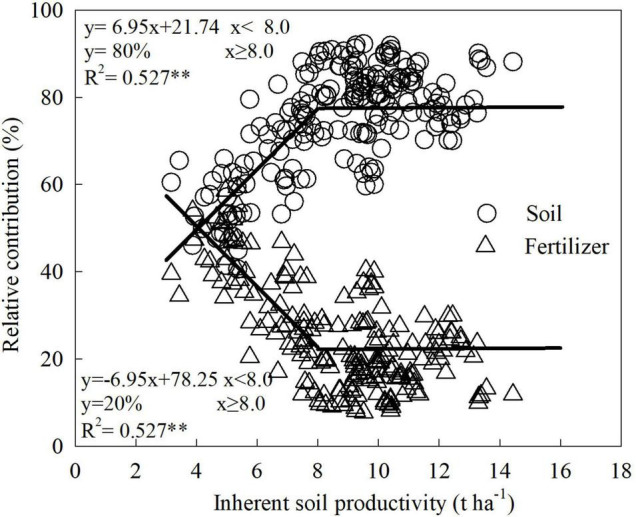
Responses of contribution of soil and N fertilizer to maize grain yield with improved soil productivity, based on the data from a 2-year field experiment conducted at five locations across large regions from the east to the west of Inner Mongolia, China. **means significant at the level of *p* < 0.01.

Why did grain yield and PFP_N_ of maize remain at a low-level when ISP was below 8 t ha^–1^? By examining the respective contribution of soil and fertilizer to grain yield, we found that the relationships between ISP and the relative contribution of soil and/or N fertilizer to grain yield could both well fit into a linear-plateau model, and the inflection point remained at 8.0 t ha^–1^ for ISP (Figure 3). However, the dynamic contribution rate of soil to grain yield along with increasing ISP was opposite to that of N fertilizer. When ISP was below 8.0 t ha^–1^, the contribution of soil to yield increased linearly by 7.0% for every 1 t ha^–1^ increase in ISP, whereas the contribution of N fertilizer to yield decreased at the same rate for each 1 t ha^–1^ ISP increase. This trade-off between soil and fertilizer was the primary cause of yield stagnating before ISP reached 8.0 t ha^–1^. After the ISP exceeded 8.0 t ha^–1^, the contribution of soil to yield stabilized at 80%, meaning that this is the basic precondition for increasing maize yield and PFP_N_ synchronously. In this scenario, the contribution of N fertilizer to maize yield dropped to a stable level of only 20% (Figure 3).

### Relationship Between Inherent Soil Productivity and Nitrogen Use Efficiency of Maize

**FIGURE 4:**
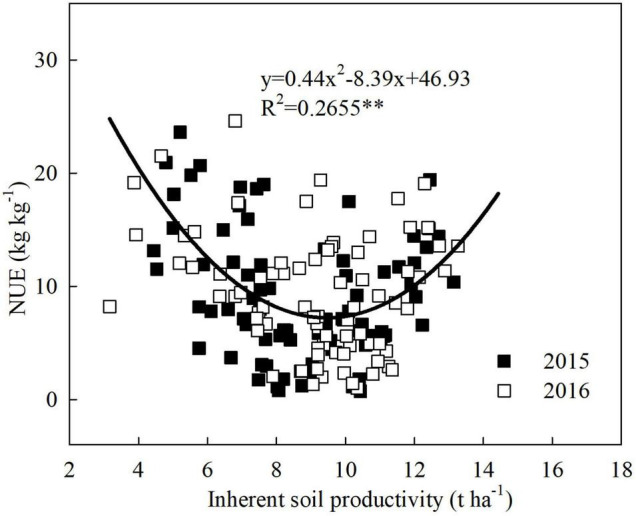
The relationship between N use efficiency (NUE) of maize and inherent soil productivity, based on the data from a 2-year field experiment conducted at five locations across large regions from the east to the west of Inner Mongolia, China. **means significant at the level of *p* < 0.01.

The NUE of maize exhibited an inverted parabolic shape along with increasing ISP, which differed notably from that of grain yield and PFP_N_ (Figure 4). When ISP was below 9.5 t ha^–1^, the NUE decreased gradually with increasing ISP. Maize NUE increased significantly only when ISP exceeded 9.5 t ha^–1^. As NUE represents the grain yield increment per unit N applied, the result showed that in the case of insufficient soil fertility, the rate of increase in the grain yield of N treatment was slower than that of ISP, indicating that soil N supply capacity might be the key limiting factor. After ISP increased to a certain level at which N supply capacity of the soil was no longer a limiting factor, ISP improvement would optimize other soil or crop growth factors, which in turn significantly enhanced the grain yield of applied N treatment and thereby resulted in a significant increase in NUE with increasing ISP.

## Discussion

### Trade-Off Between the Contribution of Soil and Fertilizer Determined a Critical Inherent Soil Productivity for Concurrent Improvement in Maize Yield and N Use Efficiency

Improving soil productivity is one of the important goals of the second “green revolution,” and it is also a main strategy to reduce the yield gap and increase resource use efficiency to support the development of green agriculture. Fan et al. (2013) proposed that improving ISP could enhance the crop yield and yield stability, while reducing input and significantly improving resource use efficiency. However, their study failed to quantify the relationships between ISP and crop yield and between ISP and NUE. To address this issue, the present study reported a multi-ecological network experiment that was conducted at five locations with large differences in ISP, from the east to the west of Inner Mongolia, China.

The results showed that there existed a critical inflection point of ISP for increasing the grain yield and PFP_N_ of maize. Below this turning point, the estimated maize yield and PFP_N_ were, respectively, 9.7 t ha^–1^ and 43.9 kg kg^–1^, which are basically comparable with the current maize yield (10.5 ± 1.6 t ha^–1^) and PFP_N_ level (40 kg kg^–1^) in China reported by Chen et al. (2014), and to the current yield (10.1 t ha^–1^) and PFP_N_ level (42.4 kg kg^–1^) of irrigated maize production in Inner Mongolia (Yang Z. et al., 2020). Therefore, our results are representative of the primary maize production regions, particularly the northeastern China.

There existed a trade-off between the contribution of soil and fertilizer to yield. Clearly, to maize yield, it was reached at ISP of 8.0 t ha^–1^. In this scenario, the contribution of soil to maize yield will reach and stabilize at approximately 80%. As Zhang (2015) reported, this 80% contribution of soil to crop yield is the current situation in Europe and North America, which is much higher than that in China with approximately 50%. For every 1 t ha^–1^ increase in ISP exceeding 8.0 t ha^–1^, the relative maize yield, actual maize yield, and PFP_N_ will increase by 7.1%, 1.2 t ha^–1^, and 4.6 kg kg^–1^, respectively. These results indicated that under low ISP levels, the dependence of maize yield on ISP was relatively weak, and increasing N fertilizer input was indispensable for increasing maize yield. In this situation, excessive N application will cause a large amount of N loss through different channels (Ma et al., 2010). Therefore, only by increasing the ISP to a certain level can maize yield and N use efficiency be improved simultaneously (Wang et al., 2014), thereby reducing the environmental load of reactive N and the carbon footprint of maize production (Ma et al., 2012). This finding is basically consistent with the conclusion of Qiao et al. (2016), who speculated that increasing ISP could not only exploit the yield potential of paddy rice, but also improve its PFP_N_ and thereby reduce N fertilizer application. Liang et al. (2018) figured out that enhancing ISP can improve crop yield and soil nutrient uptake, but reduce the recovery efficiency of fertilizer nutrients. This could be attributed mainly to the higher water and nutrient retention capacities of the fertile soils, which resulted in a higher soil mineral N content compared with less fertile soils (Zhou et al., 2019).

In our study, the average grain yield was 10% higher for IMSP than for CP, and the yield ceiling of IMSP appeared at the N uptake of 225 kg N ha^–1^, which is remarkably lower than the 254 kg ha^–1^ N uptake at the yield ceiling of CP (data not shown). Under the IMSP strategy, the highest yield was always obtained at the high plant density treatment (105,000 plants ha^–1^). In contrast, under the CP strategy, only medium- or low-plant densities could get the highest yield. This suggested that improving soil productivity could tremendously enhance the ability to support canopy productivity and population capacity with limited N consumption, i.e., enhancing N use efficiency.

### Enhancing Inherent Soil Productivity Could Significantly Reduce N Input and Reactive N Losses in Maize Production From the Long-Term Perspective

**FIGURE 5:**
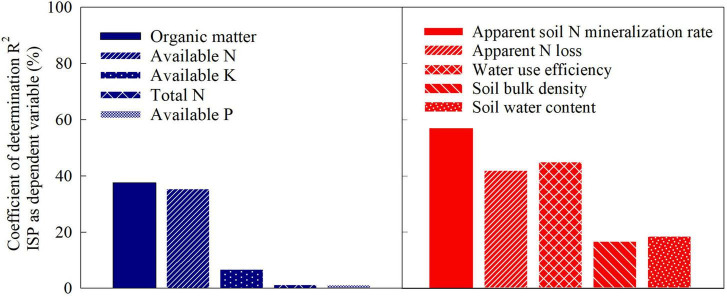
Coefficients of determination (*R*^2^) of soil physico-chemical properties in relation to inherent soil productivity of maize: data from a multisite-year network experiment conducted at five locations across large regions from the east to the west of Inner Mongolia, China.

The results of this study demonstrated that an ISP level above 9.5 t ha^–1^ was required for simultaneous increase maize yield and NUE, indicating that grain yield with N application could not be increased when the soil is not fertile enough, i.e., ISP is not high enough. An et al. (2018) speculated that the difference in the indigenous soil N source might be the primary factor driving the variation in NUE. At relatively low ISP, low N supply capacity of soil might be the core limiting factor for enhancing NUE, because more N input is needed to get equal yield increment as to improve ISP itself. When the ISP was increased to a specific level at which the soil N supply capacity was no longer a limiting factor, continuous increase in ISP might further optimize other factors of soil or crop growth, leading to significant increase in NUE along with the increased yield. Therefore, to facilitate an in-depth understanding of the ISP–NUE relationship, it is necessary to thoroughly analyze soil ecological factors that affect the dynamic change in ISP as well as physiological factors that influence maize yield potential. Liu et al. (2015) pointed out that the soil maximum water storage capacity, SOM, and soil N mineralization potential appeared to be the key factors that determine soil productivity in North China Plain. Similarly, here we identified SOM, available N, apparent soil N mineralization rate (N_mineralizable_), and soil water use efficiency (WUE) as the key determinants for ISP (Figure 5). As the core determinant, SOM content indirectly affected soil N mineralization potential, available N level, and water storage capacity, and thereby determining soil productivity (Liu et al., 2015; Farzadfar et al., 2021). For example, soils with high SOM could release more mineralizable N (Ma et al., 1999; Wu et al., 2008) and store more available water that can be absorbed by maize, thereby alleviating the negative impact of water and N stress, leading to high grain yields through increased kernel number and mean kernel weight (Liu et al., 2016). Currently, the ISP in the maize-producing regions of China is only approximately 5.6 t ha^–1^ (Fan et al., 2013), with an average contribution of only 51% to maize yield (Tang and Huang, 2008). In comparison, the contribution of soil to yield in maize belt of the United States reaches over 70% (Xu et al., 2015). This suggests that there is a substantial room for improvement in ISP in the maize-producing regions of China, or similar ecozones of the world with low ISP values.

**FIGURE 6:**
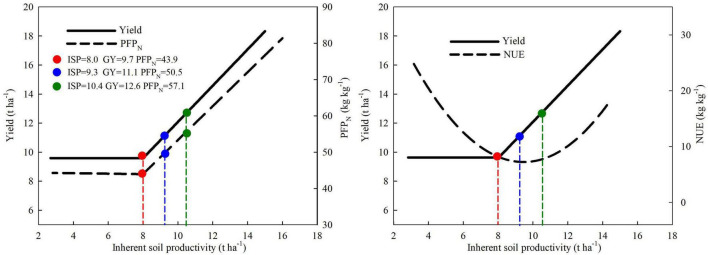
A theoretical framework for simultaneously improving grain yield and N use efficiency (NUE) of maize by enhancing inherent soil productivity.

The ISP target and corresponding soil parameters could be predicted by combining the fitted models of Figures 1, 2, 4 into Figure 6, and using the linear regression equations of ISP and soil physicochemical parameters. ISP of 8.0 t ha^–1^ was the common critical point for simultaneous increase in maize yield and PFP_N_. At this ISP level, the yield and PFP_N_ of maize were 9.7 t ha^–1^ and 43.9 kg kg^–1^, respectively. The corresponding soil parameters were 24.2 g kg^–1^ SOM, 73.2 mg kg^–1^ available N, 73.9 kg N ha^–1^ N_mineralizable_, and 20.4 kg ha^–1^ mm^–1^ WUE, with the estimated N_loss_ at a high level of 143.0 kg N ha^–1^ (Table 3). On the basis of this ISP, for targeting concurrent improvement in grain yield and PFP_N_ by 15%, i.e., achieving 11.1 t ha^–1^ of yield and 50.5 kg kg^–1^ PFP_N_, the corresponding ISP must be increased to 9.3 t ha^–1^, if all the other management strategies remain unchanged (Figure 6). Correspondingly, SOM and available N need to be increased to 26.2 g kg^–1^ and 80.8 mg kg^–1^, respectively. The N_mineralizable_ and WUE will separately reach 90.3 kg N ha^–1^ and 24.8 kg ha^–1^ mm^–1^, whereas the N losses will be reduced to 126.3 kg ha^–1^. For achieving concurrent improvement in grain yield and PFP_N_ by 30%, i.e., reaching 12.6 t ha^–1^ yield with 57.1 kg kg^–1^ PFP_N_, the corresponding ISP must be improved to 10.4 t ha^–1^ (Figure 6). In this scenario, SOM and available N should reach 28.0 g kg^–1^ and 87.2 mg kg^–1^, respectively. The N_mineralizable_ and WUE will separately reach 104.1 kg N ha^–1^ and 28.5 kg ha^–1^ mm^–1^. The N_loss_ will be further decreased to 112.2 kg N ha^–1^.

**TABLE 3:** The estimated values of soil physicochemical properties in scenarios with various maize yield and PFP_N_ targets.

Key soil factors	Unit	ISP improving goal		
		8.0 t ha^–1^	9.3 t ha^–1^	10.4 t ha^–1^
Organic matter	g kg^–1^	24.2	26.2	28.0
Available N	mg kg^–1^	73.2	80.8	87.2
Water use efficiency	kg ha^–1^ mm^–1^	20.4	24.8	28.5
N_mineralizable_	kg ha^–1^	73.9	90.3	104.1
N_loss_	kg ha^–1^	143.0	126.3	112.2

Could N input be greatly reduced by enhancing ISP? Taking the first goal of concurrent improvement in maize yield and PFP_N_ by 15% as an example, 389 kg ha^–1^ N input would be needed under the ISP of 8.0 t ha^–1^. If the ISP is at 9.3 t ha^–1^, the required N input will be reduced to 259 kg ha^–1^, which is 33% less than that under the ISP of 8.0 t ha^–1^. Similarly, taking concurrent improvement in maize yield and PFP_N_ by 30% as the goal, 577 kg ha^–1^ N input is needed under the ISP of 8.0 t ha^–1^. In contrast, if ISP is enhanced to 10.4 t ha^–1^, the N input will be reduced to 303 kg ha^–1^, i.e., 47.5% less than that of the ISP of 8.0 t ha^–1^. Obviously, enhancing ISP is essential for increasing maize grain yield while reducing N fertilizer application. As illustrated in Figure 6, if all cultivation measures except nutrient management remain unchanged, improving NUE (agronomic efficiency of N fertilizer) by increasing ISP was obviously not consistent with increasing grain yield. Taken together, our data indicates that enhancing ISP alone is not enough for improving maize yield and NUE synchronously. It is necessary to optimize other measures and explore the interactive effects of multiple measures, such as reducing N input and increasing plant density.

### Feasible Pathways for Enhancing Soil Inherent Soil Productivity

Yang et al. (2017) estimated that over the past 30 years, SOM content has been increased in the topsoil in most regions of China. The increased SOM was primarily associated with the practices of returning straw to the field and adopting conservation tillage (minimum or zero tillage) as well as the application of compost and green manure (Sharifi et al., 2014). The current amount of SOM is the long-term balance between organic matter inputs and outputs (Bingham and Cotrufo, 2016). Existing evidence shows that the amount of stabilized SOM depends on the input of organic material and its rate of oxidation, the rate at which existing SOM decomposes, soil texture, and climate. Johnston et al. (2009) pointed out that achieving significant increases in the equilibrium level of SOM in most farming systems requires very large inputs of organic matter and these have to be maintained if SOM is not to decline. Saïdou et al. (2003) and Duan et al. (2014) reported that reasonable combined application of N, P, and K fertilizer increased not only crop yield but also the N, P, and K nutrient content in the soil. Additionally, extensive research demonstrates that the long-term, continuous fertilizer application can effectively improve the physical and chemical properties of soils and then increase the nutrient and moisture content of the topsoil. This action will in turn improve soil fertility and productivity, and thereby increase crop yield (Guo et al., 2012; Qiu et al., 2014). Thus, it is necessary to adopt straw return or manure application to the field for a long period of time, along with reasonable fertilizer application, to gradually enhance soil ISP, and realize the concurrent improvement in grain yield and N use efficiency (Rusinamhodzi et al., 2011; Ciampitti and Vyn, 2014; Liang et al., 2021).

## Conclusion

Our results revealed a quantitative framework for concurrent increase in maize grain yield and NUE by enhancing ISP. Under various environmental conditions, there exists a trade-off between the contribution of soil and fertilizer to maize yield below the critical ISP of 8.0 t ha^–1^. Only by enhancing ISP beyond this critical level can both maize yield and NUE be improved simultaneously. Based on the current management strategy, if the ISP is increased to 9.3 t ha^–1^, maize grain yield and PFP_N_ can be synchronously increased by 15%, while N input and apparent N loss be reduced by 33.5 and 11.7%, respectively. A further increase in ISP to 10.4 t ha^–1^ would result in a 30% simultaneous increment in grain yield and PFP_N_, and a 47.5% reduction in N input. Meanwhile this scenario is expected to reduce the apparent N loss by 21.5%. Therefore, it is feasible to adopt a comprehensive strategy to enhance ISP and further optimize crop management measures to narrow the yield gap and increase NUE to support the development of green agriculture. In future studies, it is necessary to explore the interaction of soil properties and other agronomic management strategies and to achieve the simultaneous improvement of crop productivity and environmental sustainability by improving the intrinsic productivity of the soil, so as to cope with the new challenges brought about by global climate change.

## Data Availability Statement

The original contributions presented in the study are included in the article/supplementary material, further inquiries can be directed to the corresponding author/s.

## Author Contributions

ZW, B-LM, YL, and JG designed research. ZW, B-LM, YL, RL, QJ, XY, JS, and SH performed research. ZW and YL analyzed data. ZW, B-LM, YL, and JG wrote the manuscript. All authors contributed to the article and approved the submitted version.

## Conflict of Interest

The authors declare that the research was conducted in the absence of any commercial or financial relationships that could be construed as a potential conflict of interest.

## Publisher’s Note

All claims expressed in this article are solely those of the authors and do not necessarily represent those of their affiliated organizations, or those of the publisher, the editors and the reviewers. Any product that may be evaluated in this article, or claim that may be made by its manufacturer, is not guaranteed or endorsed by the publisher.
